# Time Trend of Overweight and Obesity in Adults from Rio Branco, Acre, Western Brazilian Amazon (2006–2020)

**DOI:** 10.3390/nu14040742

**Published:** 2022-02-10

**Authors:** Flávia Santos Batista Dias, Yara de Moura Magalhães Lima, Fernanda Andrade Martins, Mônica da Silva-Nunes, Andréia Moreira de Andrade, Alanderson Alves Ramalho

**Affiliations:** 1 Graduate Program in Public Health, Federal University of Acre, Rio Branco 69920-900, AC, Brazil; flavia.dias@ufac.br (F.S.B.D.); yara.moura@sou.ufac.br (Y.d.M.M.L.); andreia.andrade@ufac.br (A.M.d.A.); 2 Center for Health Sciences and Sports, Federal University of Acre, Rio Branco 69920-900, AC, Brazil; fernanda.martins@ufac.br; 3 Department of Medicine, Center for Biological and Health Sciences, Federal University of São Carlos, São Carlos 13565-905, SP, Brazil; monicamamtra@gmail.com

**Keywords:** obesity, overweight, nutrition surveys, epidemiological surveys, time series studies

## Abstract

This study aimed to analyze overweight trend and obesity in adults from Rio Branco, Acre, Western Brazilian Amazon, from 2006 to 2020. This is a time series study, with data from Surveillance System for Risk and Protective Factors for Chronic Diseases by Telephone Survey (VIGITEL). To estimate annual percentage change (APC) and 95% confidence intervals, the software Jointpoint Regression Analysis v.4.6.0.0., was used. In Rio Branco, overweight prevalence ranged from 44.0% in 2006 to 58.9% in 2020, with a bigger frequency among men than that among women. Obesity prevalence has increased from 12.5% in 2006 to 21.4% in 2020, similar between both sexes. From 2006 to 2020, overweight APC was 5.2% (95% CI: 1.4; 9.1) by 2010, and decreased to 1.3% by 2020. Public policies to control obesity and its risks must be both, implemented as strengthened.

## 1. Introduction

Being overweight and obesity are considered problems of global public health. The World Health Organization (WHO) has estimated an almost three-times increase in obesity’s prevalence in the last four decades (between 1975 and 2016). In 2016, more than 1.9 billion adults were overweight. More than 650 million of them were obese, about 13% of world population (11% of men and 15% of women) [1].

Evidence indicates that the amount of overall death and disability-adjusted life years (DALYs) attributable to obesity considerably increased between 1990 and 2017. Globally, in 2017, obesity contributed to 34.1 million deaths and 1.2 billion DALYs. The increasing of mortality and DALYs associated with these risk factors was substantial for both sexes in 2017, with 2.4 million deaths and 70.7 million DALYs in women and 2.3 million deaths and 77.0 million of DALYs in men, reflecting overweightness and its vast disease burden. Cardiovascular diseases were the main causes of DALYs, followed by diabetes, kidney diseases, and neoplasms (representing 89.3% of all DALYs associated with overweight and obesity) [2,3].

The increasing of the prevalence of obesity is a reality not only for high-income countries. Since the 2000s, a growing trend has been shown in low and middle-income countries [1]. Regional differences in diet and health quality are associated with socioeconomic conditions. According to the Latin American Health and Nutrition Study (ELANS), people with low socioeconomic conditions consume less fresh and/or minimally processed food than individuals in better situations. Thus, poor diet and health quality, such as in abdominal obesity, are associated with socioeconomic conditions of the respondents [4].

Latin America and the Caribbean are showing increasing trends of obesity. Among Latin countries, one of the highest prevalence was observed in Brazil. The Surveillance System for Risk and Protective Factors for Chronic Diseases by Telephone Survey (VIGITEL) observed that in men there was an increasing trend (from 47.5% to 57.3% between 2006 and 2017), and in women the prevalence of obesity increased from 38.5% to 51.2% in the same period. The largest increases in obesity prevalence were observed in populations of capital cities in the North, Northeast, and Midwest of Brazil [5,6,7].

Rio Branco is the capital city of the state of Acre, located in the Brazilian Amazon Valley. Despite its food biodiversity, it has one of the highest rates of obesity prevalence in Brazil. From 2006 to 2016, there was an 8.7% increase in the average body mass index (BMI) among men (from 25.3 kg/m^2^ to 27.5 kg/m^2^), about 0.16 kg/m^2^ per year. In 2017, Rio Branco city had one of the highest morbid obesity prevalence (3.4%) of all Brazilian capital cities [5,7,8].

Thus, the aim of this study is to analyze time trend of overweight and obesity in adults from Rio Branco, Acre, and the Western Brazilian Amazon from 2006 to 2020.

## 2. Materials and Methods

A population-based observational time series study was designed, using as its data source the results from 2006 to 2020 of the Surveillance System for Risk and Protective Factors for Chronic Diseases by Telephone Survey (VIGITEL) for Rio Branco, Acre, in the Western Brazilian Amazon.

Rio Branco city is located in the Northern Region of Brazil and it concentrates 47.32% of the entire population of the state of Acre; around 89.42% of its population are located in urban areas. VIGITEL used probability samples of the adult population (18 years old or older) living in the capital cities of the states of Brazil.

The sampling procedures employed by VIGITEL system aim to obtain probabilistic samples from the population of adults living in households with at least one landline telephone in the year. That system establishes a minimum sample size of 2000 individuals aged 18 or older, in order to estimate with a 95% confidence interval and a maximum error of about two percentage points to the frequency of risk factors for chronic diseases in the adult population. The maximum errors expected are of about three percentage points for the specific estimates by sex, assuming similar proportions of men and women in the sample. In each active residential landline, where there was contact with an adult resident and agreement to participate in the study, the resident selected randomly for the interview. The survey collected, among other information, self-reported weight and height, which enabled the diagnosis of the anthropometric status of the respondent by means of the body mass index, and the estimates of prevalence of overweight and obesity in the adult population of Rio Branco [5].

Of the 27,153 interviewees throughout the 15 years of the survey in Rio Branco, 3231 individuals were excluded, of which 2948 (10.86%) were excluded due to lack of information on weight or height and 283 (1.04%) were pregnant women or women who did not know if they were pregnant at the time of the interview. As dependent variables, in this study we used either being overweight and being obese. Body mass index (BMI) ≥25 kg/m^2^ was considered overweight and BMI ≥ 30 kg/m^2^ obesity [9], calculated from the weight in kilograms divided by the square of the height in meters, both self-reported, according to the questions: “Do you know your weight (even if it is an approximate value)?”, “Sir/Mistress, do you know what’s your height?”. Regarding the percentage of adults with obesity, we have the number of individuals with obesity/number of individuals interviewed. Also, in the questions referred to, for the purpose of selection of information, only individuals who reported weight >30 kg and <300 kg, and regarding to height >1.20 m and <2.20 m) are accepted [5].

Independent variables were: sex (male/female); age group (18 to 24 years/25 to 34 years/35 to 44 years/45 to 54 years/55 to 64 years/65 or older); skin color (white/non-white); level of schooling (0 to 8 years/9 to 11 years/12 years or more); marital status (no partner/companion); regular consumption of fruits, vegetables and legumes (no/yes); excessive consumption of sugary drinks (no/yes) and TV time per day in five days (do not watch or watch up to 3 h/watch three or more hours).

The Jointpoint Regression Analysis Software v.4.6.0.0 (The National Cancer Institute, MD, USA) was used for the time series analysis. The software allows us to detect linear patterns in the distribution of the estimates of interest over two or more time intervals, identifying segments that have specific trends in estimates using Annual Percentage Change (APC), which is the average of the relative percentage changes between each of the fifteen time intervals. In this analysis, the null hypothesis is given by a linear equation, while the alternative refers to a linear model with two or more segments that better explains the variability of the data, taking into account the value of *p* < 0.05.

Since this research used data of public use and access, made available by Brazilian Ministry of Health in an unrestricted way and without nominal identification, that research waives ethical appreciation in the terms of the National Health Council Resolution (CNS 466/12), which gives provisions for research involving human beings in Brazil.

## 3. Results

The sample of this study was composed of valid interviews conducted by telephone survey with 23,922 individuals aged 18 or older, from 2006 to 2020 in the city of Rio Branco, Acre.

### 3.1. Overweight and Obesity Prevalence

In Table 1, the distribution of overweight prevalence (individuals with BMI ≥ 25 kg/m^2^) according to socio-demographic characteristics is represented. Overweight prevalence in individuals aged 18 years and older, in the first survey year (2006) was 44.0%, and in the last year (2020), it was 58.9%, with the highest prevalence in 2016, with 61.3%. Regarding sex, men had higher prevalence than women had; also reaching the highest frequency in 2016, with 66.4% for men and 56.5% for women in 2018.

Obesity prevalence (individuals with BMI ≥ 30.0 kg/m^2^) in adults in Rio Branco, increased from 12.5% in 2006 to 21.4% in 2020, showing a higher frequency in 2016: 23.9% (Table 2). When stratifying by sex, prevalence was similar in both men and women. In females, the prevalence in 2006 was 12.9%, and in 2020, it was 20.6%, reaching a higher frequency in 2016: 22.8%. In men, prevalence of obesity rose from 12.1% in 2006 to 22.3% in 2020, reaching the highest prevalence with 25.1% in 2019. Regarding age and education, the highest prevalence was in individuals between 35 and 64 years old who had no or up to eight years of education for both overweight and obesity.

### 3.2. Time Trend of Overweight Prevalence

**Figure 1:**
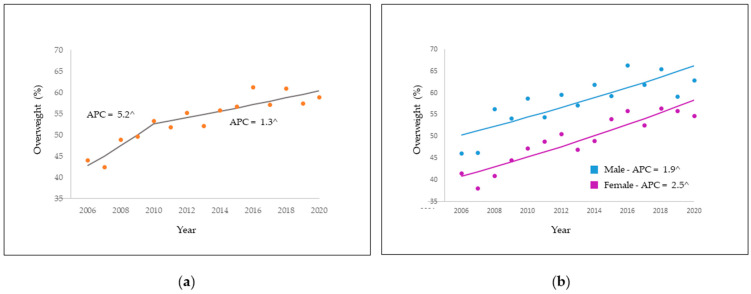
Overweight prevalence trend (**a**) and overweight trend by sex (**b**). Rio Branco, AC, Brazil, from 2006 to 2020. ^ APC is significantly different from zero at the alpha = 0.05 level.

From 2006 to 2020, overweight variance was 5.2% per year (95% CI: 1.4; 9.1) until 2010, with a reduction of 1.3% per year until the end of the analyzed period; however, it is significant for the whole period, as represented in Figure 1.

It was observed that the most consistent upward trends occurred among females (APC: 2.5%; 95% CI: 1.8; 3.2), in those of the 18–24 and 25–34 age groups, by 2.7% per year (95% CI: 0.8; 4.8; 1.4; 4.0, respectively), and level of schooling between 9 and 11 years (APC: 5.4%; 95% CI: 3.0; 7.8). Regarding marital status, annual variation in the period was greater in those who had no partner, by 2.7% per year (95% CI: 1.7; 3.7), and in those who had self-reported white skin color, by 2.3% per year (95% CI: 1.4; 3.1). Regarding lifestyle, in individuals who reported watching three or more hours of television on at least 5 days a week, trend was upward throughout the period, by 3.0% per year (95% CI: 1.8; 4.1). And, still with an ascendency about the regular consumption of fruits, vegetables and greens on five or more days in the week 2.2% per year (95% CI: 1.4; 3.0). Regarding the non-consumption of soft drinks and artificial juices on five or more days in the week was 2.0%/year (95% CI: 1.5; 2.6) during such studied period (Table 3).

### 3.3. Time Trend of Obesity Prevalence

**Figure 2:**
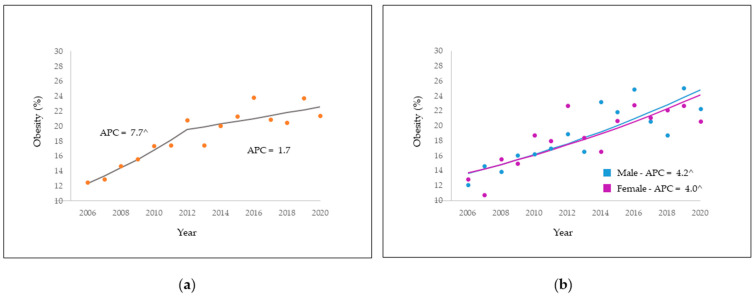
Obesity prevalence trend (**a**) and obesity by sex (**b**). Rio Branco, AC, Brazil, from 2006 to 2020. ^ APC is significantly different from zero at the alpha = 0.05 level.

The analysis of time trend of obesity prevalence shows a significant upward increase of 7.7% per year (95% CI: 4.5; 10.9), until 2012. This variation underwent a considerable decrease, starting in 2012, of 1.7% per year (95% CI: −0.2; 3.7) until the end of the studied period, but it did not show statistical significance, as shown in Figure 2.

Regarding socio-demographic characteristics, the most robust trends were found in males, with 4.2%/year (95% CI: 2.7; 5.6), in those aged 25 to 34 years old (5.5%/year—95% CI: 2.7; 8.4), and those aged 65 years and older 5.0%/year (95% CI: 2.6; 7.4), who had 9 to 11 years of schooling, 6.4%/year (95% CI: 4.5; 8.3). Participants who responded white to self-report skin color, the annual rate over the period was 4.7%/year (95% CI: 2.0; 5.7), and was highest in those who had no partner, at 4.8%/year (95% CI: 2.6; 7.1). The most consistent upward trends in obesity observed in lifestyle variables for the entire period, are found in those who self-reported regular consumption of fruits, vegetables, and greens 5.2%/year (95% CI: 3.1; 7.4). In individuals who did not consume soft drinks or sugary beverages, 4.7%/year (95% CI: 1.6; 7.9), and for those who watch three or more hours of television on five days per week, 6.2%/year (95% CI: 4.0; 8.4) for the entire period under study (Table 4).

## 4. Discussion

In the last 15 years, obesity prevalence has increased considerably in Rio Branco, Acre, for both sexes. Trends of being overweight and obesity prevalence were upward throughout the analyzed period. However, we observed a deceleration of this growth in the last decade.

Overweight and obesity prevalence has shown an increase in the averages of other Brazilian capital cities. According to VIGITEL (in 2016 and 2019), frequency of overweight adults increased in some Brazilian capitals, such as: Florianópolis, from 48.8% (95% CI: 45.3; 52.2) to 53.6% (95% CI: 50.4; 56.8); Goiânia, from 48.5% (95% CI: 45.6; 51.4) to 52.7% (95% CI: 49.6; 55.8); Manaus, from 56.3% (95% CI: 52.7; 59.8) to 60.9% (95% CI: 57.5; 64.4); Porto Alegre, from 54.9% (95% CI: 52.0; 57.8) to 59.2% (95% CI: 56.0; 62.3); Recife, from 55.6% (95% CI 52.8; 58.5) to 59.5% (95% CI 56.5; 62.5) and Federal District, from 48.8% (95% CI: 44.9; 52.8) to 55.0% (95%CI: 51.2;58.9). Obesity also showed an increase in adults in Brazilian capital cities, such as: Belo Horizonte, from 16.6% (95% CI: 14.7; 18.5) to 19.9% (95% CI: 17.7; 22.2); Boa Vista, from 18.7% (95% CI: 15.5; 21.8) to 21.2 (95% CI: 16.8; 25.5); Florianópolis, from 14.5% (95% CI: 12.2; 16.8) to 17.8% (95% CI: 15.5; 20.1); Goiânia, from 16.3% (95% CI: 14.1; 18.5) to 19.5% (95% CI: 17.1; 21.8); São Luís, from 15.6% (95% CI: 13.2; 18.1) to 17.2% (95% CI: 14.2; 20.1) and Federal District, from 16.7% (95% CI:13.6; 19.7) to 19.6% (95% CI: 16.3; 22.8) [10,11].

Similar to Brazil, obesity frequency has also been a reality in other countries. United States has shown a considerable increase in obesity prevalence since 1999, despite regional differences. In 2015–2016, the adult population showed a considerable prevalence of obesity and overweight in both genders; the frequency of obesity and being overweight among men were 33.7% and 74.7%, and among women 41.5% and 68.9%, respectively. In Southern Africa, a global study of disease burden, injury, and risk factors was conducted from 1990 to 2019, making it possible to analyze that among developing countries, South Africa 44.7% (95% CI: 42.5; 46.8), Swaziland 33.9% (95% CI: 31.7; 36.0), and Lesotho 31.6% (95% CI: 29.8; 33.5) had the highest prevalence of obesity in 2019 [12,13].

A meta-analysis evaluated overweight time trend in Brazilian adults between the 1970s and 2020s, and estimated that the combined prevalence of overweight individuals increased from 33.5% (95% CI: 25.0; 42.6%) in 1974–1990 to 52.5% (95% CI: 47.6; 57.3%) in 2011–2020. From 1974 to 1990, it was 24.6% (95% CI: 18.8; 31.0%) and 40.5% (95% CI: 37.0; 43.9%) from 2011 to 2020. The combined prevalence of obesity in Brazilian adults increased by 15.0% from 1974 to 1990 and from 2011 to 2020. The increases were observed for both, men and women, in almost all periods. Being overweight and obesity prevalence remained higher among women in all periods [14].

Gomes et al. [15] observed similar results to our study, when estimating obesity and being overweight trends from 2002 to 2013 by sex, age, and schooling level among Brazilian adults. There were analyzed nationwide surveys: the 2002/2003 and 2008/2009 Family Budget Surveys (POF) and the 2013 National Health Survey (PNS). Obesity prevalence increased from 7.5% to 17.0%, from 2002 to 2013 among adults between the ages 20 and 39 years old, and from 14.7 to 25.7% among those between 40 to 59 years old, slightly higher among young women. In each survey, level of schooling was positively associated with obesity prevalence among men, while this association was negative among women. The largest increase in obesity prevalence was 90% (11.9 to 22.5%) and occurred from 2008 to 2013 among women with a middle level of education, while at the pre-primary level there was a 42% increase (20.4 to 29.0%) [15].

In a study with adult population residing in Macapá, in the Northern Region of Brazil, from 2006 to 2018, it was possible to verify a higher trend, if comparing to that study, of an increasing, distinguishable by gender. Between 2006 and 2018 obesity prevalence for both sexes increased from 13.9% to 20.1% (6.2% per year); in males the increase was from 16.4% to 17.8% (1.4% per year) and in females from 11.6% to 22.2% (10.6% per year). Another research analyzed trends in the overweight and obesity prevalence in the state of Espírito Santo, Southeast Region of Brazil, between 2009 and 2018 with data from the Food and Nutrition Surveillance System (SISVAN). We can observe a trend of increasingly overweight among adult men (20 to 59 years old), from the north and south of the state and adult women (20 to 59 years old) from all regions of the state. However, women living in the metropolitan area showed the greatest upward trend with APC of 7.3% (95% CI 7.2; 7.4) [16,17].

In the Southern Region of Brazil, the Pelotas cohort is also in line with the results of our study. The authors analyzed obesity prevalence in adolescence and adulthood in individuals belonging to the 1982 through 2012 birth cohort, according to social and demographic characteristics. Being overweight and obesity prevalence was lower in women with higher socioeconomic status. However, the increase in overweight was also greater in women with low socioeconomic status and at the age of 30. Among men aged 18 to 30 years old, regardless of age, skin color was not associated with being overweight and obesity, and the difference in prevalence decreased when comparing men of lower and higher socioeconomic status. Among women, skin color was associated with obesity at ages 23 and 30 years [18].

By investigating the variation of anthropometric indicators from 2013 to 2019, as well as the factors associated with obesity in Brazil, using information from the National Health Survey, Ferreira et al. [19] estimated a significant increase in obesity prevalence, from 20.8 to 25.9%. They found that the greatest variations occurred in males of the 40 to 59 age group (9.1%). They also found that, among women, the largest increases occurred among those with low education (8.7%) and non-white women (6.0%). Both sexes were associated with obesity, age, and marital status. Regarding education level, it was directly associated among men and inversely associated among women [19].

When comparing the results of our study with international studies, they observed sharp differences between sexes, with more fluctuations among men. Yang et al. [20] conducted a survey of gender-specific time trends in the prevalence of overweight among Chinese adults from 2008 to 2015. Overweight tended to increase until middle age and decrease thereafter. When compared to women, overweight prevalence was significantly higher for men, but this pattern reversed after age 60. Individuals with a high level of schooling had a consistently higher overweight prevalence than their peers, with low education level [20]. In the United States, the National Health and Nutrition Examination Survey (NHANES) is conducted every two years by the National Center for Health Statistics and funded by the Centers for Disease Control and Prevention. The survey measures most recent obesity prevalence and national trends over time, including by age group, gender, and race, with data available through 2017–2018. According to NHANES, there was a significant upward trend in adult obesity prevalence from 1999 to 2000 and from 2017 to 2018. There was also no statistically significant change in adult obesity frequencies from 2015 to 2016 and from 2017 to 2018. Also, according to the most recent NHANES, there was no significant difference between men and women or between age groups (20 to 60 years and older), however, for those who self-reported to be black, obesity prevalence was higher if compared to other skin colors and ethnic groups [21].

Brazil follows the trends of the other Latin American and Caribbean countries, with obesity on the rise. The increasing urbanization and rising socioeconomic status of these countries, coupled with a nutritional transition, decreasing malnutrition, consumption of family farm foods, increasing physical inactivity, and consumption of ultra-processed foods, has contributed to these trends continuing to grow [6].

The significant increase in obesity in Rio Branco, from 2006 to 2010 may be associated with economic, social, and demographic changes in Brazil that may have reflected in changes of eating habits [22]. During that period, there was an increasing trend of the Brazilian purchasing power, with growth rates of gross domestic product (GDP) ranging from 4.2% to over 5%. In addition to this, there was an accelerated urbanization and an increase in family income, as well as an increase in spending with food outside home, based on the consumption of fast food and ultra-processed food [23,24,25]. These changes can be observed specifically in Rio Branco by the expansion and construction of new highways in this period, the opening of the first mall in the state, as well as the first national and international fast-food franchises in the city.

During 2011/2012, despite remaining upward, a decrease in the annual percentage change in the trend of overweight and obesity in Rio Branco, is observed. The results of the Family Budget Surveys (POF) between the 2002–2003 and 2008–2009 editions, suggest an upward course regarding family expenses. The economic crisis reached Brazil around 2014 and dampened previously growing trends, such as the increase in out-of-home food expenditures, as well home spending on meat, offal, and fish. In addition, since 2011 there has been an intensification of public policies to fight obesity in Brazil, such as the National Food and Nutrition Policy (PNAN), aiming to guarantee the right of promoting health eating practices [26]. However, with an economic slowdown, there has been a rise in the consumption of ultra-processed category and sugar has again become more consumed according to the findings of the latest 2017–2018 POF survey. [23].

This study also assessed the time trend related to other socio-demographic and lifestyle factors. Among them, an increase in this variation was observed in those who self-reported to be white and in those who self-reported to watch three or more hours a day of television on up to five days a week for those who are overweight. The observations of trend for marital status (e.g., companion or no partner) showed no statistically significant differences between responses, considering their confidence intervals at 95%. Similar findings were found by Reis et al. [27], in Belo Horizonte, regarding the recommended consumption of fruits and vegetables, indicating an increase of 1.05%/year, ranging from 23% in 2008 to 31.1% in 2016. He further found a reduction in the prevalence of regular consumption of soda/sweetened beverages (≥five days/week) by 1.89%/year, ranging from 35.3% in 2007 to 15.2% in 2016, which in our study showed the same variation for a trend in consumption over the period. These variations seem divergent from the point of view of overweight, but considering the reverse causality hypothesis, it is not possible to determine cause and effect for these events in cross-sectional studies. Vaz and Hoffmann [23] reinforce the hypothesis by pointing out that there has been a reversal in the trend of increasing household spending on meat, offal and fish, as well as a decrease on spending on all categories of diet products from 2008 to 2017.

Similar to being overweight, the trend toward obesity has shown to be greater in self-reported white individuals and in those who reported watching three or more hours of television per day on up to five days of the week. Regarding lifestyle in the obese population, the annual trends for regular consumption of fruits, vegetables, and legumes were more expressive, as well as for excessive consumption of sugary drinks on five or more days a week, during the entire period of the historical series. Changes in a population’s eating pattern can be attributed to several issues, as raised by Vaz and Hoffmann [24], when they propose that the economic crisis that hit the country from mid-2014 brought about a change in this scenario, with an increase in the number of unemployed, discouraged and inactive workers, whose income from work is nil. According to him, these inequalities in the per capita distribution of family income experienced a decline between 2001 and 2015, and increased again in the following three years. The author also states that, after a large reduction from 2003 to 2014, poverty grew considerably from 2015 to 2017, and that for many researchers, this corroborates that the growth model driven by household consumption has been suppressed (even though a high percentage of Brazilian families have not reached a consumption standard that guarantees food security and satisfaction of other basic needs).

This study may present possible limitations associated with information bias. The collection of self-reported data may reflect inaccuracies in the BMI calculation. However, the methodology employed by VIGITEL is widely used and recommended in health surveys [28,29], besides be validated for Brazil [29]. It should be noted that, in this study, only the individuals who had the weight and height data were analyzed. Other limitations to consider include some data, such as per capita income and type of housing, not included in the instrument used by VIGITEL (which do not allow for a detailed breakdown of the socio-economic status of the participants).

However, the data used in this study are representative for the population of Rio Branco, aged 18 years or older, and it gives strength to this study. Furthermore, analyses of current trends in obesity in adults in the Amazon and other low and middle-income regions have been scarce. Thus, this study can complement others, contributing to a better monitoring of obesity.

## 5. Conclusions

In Rio Branco, overweight prevalence ranged from 44.0% in 2006 to 58.9% in 2020. Obesity prevalence has increased from 12.5% in 2006 to 21.4% in 2020. From 2006 to 2020, overweight APC was 5.2% (95% CI: 1.4; 9.1) by 2010, and decreased to 1.3% by 2020. This study can complement others, contributing to a better monitoring of obesity. Public policies to control obesity and its risks must be both, implemented as strengthened.

## Figures and Tables

**Table 1:** Distribution of overweight prevalence (%) according to sex, skin color, age, and education in adults over 18 years of age. Rio Branco, Acre, from 2006 to 2020.

**Year**	**2006**	**2007**	**2008**	**2009**	**2010**	**2011**	**2012**	**2013**	**2014**	**2015**	**2016**	**2017**	**2018**	**2019**	**2020**
**N^a^**	1751	1768	1796	1787	1771	1768	1467	1757	1340	1707	1622	1642	1301	1576	869
**N^b^**	15,472	17,142	16,150	16,352	18,539	19,352	19,519	20,419	20,255	20,126	21,681	21,972	22,103	21,835	22,123
Total	44.0	42.5	48.9	49.6	53.3	51.8	55.2	52.1	55.8	56.7	61.3	57.2	61.0	57.5	58.9
**Sex**															
Male	46.2	46.3	56.3	54.2	58.8	54.5	59.7	57.2	61.9	59.3	66.4	61.9	65.5	59.2	63.0
Female	41.6	38.1	41.0	44.6	47.3	48.9	50.6	47.0	49.0	54.0	55.9	52.6	56.5	55.9	54.8
**Age group (in years)**															
18–24	25.4	20.7	27.2	30.6	26.6	30.1	38.8	37.1	33.6	29.0	37.9	31.8	39.8	39.0	30.3
25–34	42.9	39.9	44.7	48.0	59.9	48.0	57.4	48.4	57.4	61.2	59.3	55.7	63.5	55.1	63.8
35–44	52.1	54.7	59.4	59.3	60.4	67.1	59.7	58.4	60.5	63.7	69.9	67.2	66.6	64.9	63.2
45–54	58.6	55.6	72.1	61.5	60.9	65.3	66.3	63.0	67.2	66.6	78.1	66.6	70.0	68.2	68.4
55–64	59.0	62.8	62.5	51.8	70.1	60.8	55.8	60.3	71.3	65.8	67.6	66.8	66.6	64.1	58.5
65 or more	50.3	48.9	52.7	65.2	55.3	53.2	54.5	68.7	53.5	60.7	63.6	65.3	62.5	58.5	65.0
**Skin color**															
White	42.7	42.1	52.9	51.0	49.7	47.8	54.6	52.1	57.5	55.7	59.7	56.0	55.1	58.3	64.0
Non-white	45.0	57.9	47.3	49.0	54.7	53.0	53.8	51.0	53.8	56.5	61.7	57.2	63.4	57.1	57.3
**Level of schooling (by years of studying)**															
0–8	50.6	46.8	58.8	55.5	61.8	57.6	61.5	59.0	62.9	62.1	69.2	67.8	65.5	62.7	57.9
9 a 11	38.2	37.9	38.1	44.7	47.2	47.0	50.9	49.9	53.6	53.3	59.6	55.1	60.7	56.7	59.3
12 or more	36.1	38.7	43.7	44.1	44.6	48.7	50.8	44.7	48.0	54.0	54.5	49.4	57.7	54.2	59.1

N^a^ = number of valid interviews excluding pregnant women, suspected pregnant women and individuals without complete information. N^b^ = expanded sample.

**Table 2:** Distribution of obesity prevalence (%) according to sex, skin color, age, and education in adults over 18 years of age. Rio Branco, Acre, from 2006 to 2020.

**Year**	**2006**	**2007**	**2008**	**2009**	**2010**	**2011**	**2012**	**2013**	**2014**	**2015**	**2016**	**2017**	**2018**	**2019**	**2020**
**N^a^**	1751	1768	1796	1787	1771	1768	1467	1757	1340	1707	1622	1642	1301	1576	869
**N^b^**	15,472	17,142	16,150	16,352	18,539	19,352	19,519	20,419	20,255	20,126	21,681	21,972	22,103	21,835	22,123
Total	12.5	12.9	14.7	15.6	17.4	17.5	20.8	17.5	20.1	21.3	23.9	20.9	20.50	23.8	21.4
**Sex**															
Male	12.1	14.6	13.9	16.1	16.2	17.0	18.9	16.6	23.2	21.9	24.9	20.6	18.80	25.1	22.3
Female	12.9	10.8	15.6	15.0	18.8	18.0	22.7	18.4	16.6	20.7	22.8	21.1	22.10	22.7	20.6
**Age group (in years)**															
18–24	7.0	6.9	4.8	7.1	5.2	8.8	12.9	7.9	5.0	8.3	12.4	8.3	4.70	10.3	13.7
25–34	8.7	10.6	13.4	11.6	17.6	13.3	19.1	15.0	21.7	22.7	22.3	13.7	20.00	24.2	20.4
35–44	15.9	16.0	22.5	23.2	21.7	25.2	23.2	24.5	25.0	24.4	27	31.7	23.40	31.7	21.4
45–54	19.0	15.1	22.3	24.7	22.0	26.7	29.9	22.4	21.6	29.5	31.8	27.9	27.10	26.4	22.5
55–64	25.8	27.0	19.7	15.9	32.3	21.7	24.4	20.2	31.1	22.8	32.6	29.1	33.80	26.8	30.3
65 or more	13.8	16.1	10.4	20.9	17.8	15.9	20.0	23.6	23.5	22.6	25.1	26.2	23.20	21.7	29
**Skin color**															
White	11.4	12.9	14.1	16.6	18.9	15.7	26.3	15.5	17.9	23.3	20.7	18.9	16.50	26.2	28.7
Non-white	12.9	11.3	14.9	15.3	17.0	18.0	16.9	17.5	20.0	20.8	24.8	21.2	21.90	22.7	19.1
**Level of schooling (in years of studying)**															
0–8	14.5	15.1	20.0	20.4	20.8	22.2	24.7	22.2	26.3	25.1	31.1	26.4	25.2	32.3	21.9
9 a 11	11.3	9.8	9.0	11.4	15.7	15.6	18.7	14.7	16.9	18.8	21.4	19.2	22.8	22.2	22.4
12 or more	8.9	12.2	11.6	11.4	12.7	11.5	17.0	14.1	15.4	19.7	18.9	17.4	14.0	18.7	20.2

N^a^ = number of valid interviews excluding pregnant women, suspected pregnant women and individuals without complete information. N^b^ = expanded sample.

**Table 3:** Distribution of the annual percentage change (APC) of overweight in adults over 18 years old. Rio Branco, Acre, from 2006 to 2020.

	%				
	**2006**	**2020**	**APC**	**95% CI**	**Period**
Total	44.1	58.9	5.2 ^	1.4; 9.1	2006–2010
			1.3 ^	0.4–2.2	2010–2020
**Sex**					
Male	46.2	63	1.9 ^	1.0; 2.7	2006–2020
Female	41.7	54.8	2.5 ^	1.8; 3.2	2006–2020
**Age group** (**in years**)					
18–24	25.4	30.3	2.7 ^	0.8; 4.8	2006–2020
25–34	42.9	63.8	2.7 ^	1.4; 4.0	2006–2020
35–44	52.2	63.2	1.3 ^	0.6; 2.0	2006–2020
45–54	58.6	68.4	1.0 ^	0.2; 1.8	2006–2020
55–64	59	58.5	0.5	−0.6; 1.7	2006–2020
65 or more	50.3	65	1.5 ^	0.4; 2.7	2006–2020
**Skin color**					
White	41.7	64	2.3 ^	1.4; 3.1	2006–2020
Non-white	45	57.3	1.4 ^	0.5; 2.3	2006–2020
**Level of schooling** (**in years of studying**)					
0–8 years	50.7	57.9	2.6 ^	1.1; 4.2	2006–2017
			−4.8	−13.5; 4.8	2017–2020
9–11 years	38.2	59.3	5.4 ^	3.0; 7.8	2006–2012
			2.1 ^	0.6; 3.7	2012–2020
12 years	36.1	59.1	2.9 ^	2.0 a 3.7	2006–2020
**Marital status**					
No partner	33.5	49.6	2.7 ^	1.7; 3.7	2006–2020
Companion	54	68.7	2.0 ^	1.4 a 2.7	2006–2020
Regular consumption of fruits and vegetables *					
No	43.4	58.1	2.2^	1.4; 3.0	2006–2020
Yes	47	61.6	1.9^	1.3 a 2.4	2006–2020
Excessive consumption of sugary drinks **					
No	43.7	59.3	2.0 ^	1.5; 2.6	2006–2020
Yes	45	56.6	1.9 ^	0.5; 3.3	2006–2020
**TV time per day on 5 days a week**					
Does not watch or watches up to 3 h	44.4	55.5	1.8^	1.1; 2.6	2006–2020
Watches 3 or more hours	43.1	70.4	3.0^	1.8; 4.1	2006–2020

* Regular consumption of fruits, vegetables and legumes on five or more days a week. ** Consumption of soft drinks and artificial juices with sugar on five or more days a week. ^ APC is significantly different from zero at the alpha = 0.05 level.

**Table 4:** Distribution of the annual percentage change (APC) of obesity in adults over 18 years. Rio Branco—AC, from 2006 to 2020.

	%				
	**2006**	**2020**	**APC**	**95% CI**	**Period**
Total	12.5	21.4	7.7 ^	4.5; 10.9	2006–2012
			1.7	−0.2; 3.7	2012–2020
**Sex**					
Male	12.1	22.3	4.2 ^	2.7; 5.6	2006–2020
Female	12.9	20.6	4.0 ^	2.1; 5.9	2006–2020
**Age group** (**in years**)					
18–24	7.0	13.7	2.9	−1.6; 7.5	2006–2020
25–34	8.7	20.4	5.5 ^	2.7; 8.4	2006–2020
35–44	15.8	21.4	2.9 ^	0.9; 5.0	2006–2020
45–54	19.0	22.5	2.3 ^	0.1; 4.5	2006–2020
55–64	25.8	30.3	2.2	−0.5; 4.9	2006–2020
65 or more	13.8	29.0	5.0 ^	2.6; 7.4	2006–2020
**Skin color**					
White	11.4	28.7	4.7 ^	2.0; 7.4	2006–2020
Non-white	12.9	19.1	4.0 ^	2.7; 5.4	2006–2020
**Level of schooling** (**in years of studying**)					
0–8 years	14.4	21.9	3.8 ^	2.0; 5.7	2006–2020
9–11 years	11.3	22.4	6.4 ^	4.5; 8.3	2006–2020
12 or more	8.9	20.2	4.6 ^	2.8; 6.5	2006–2020
**Marital status**					
No partner	8.8	19.2	4.8^	2.6; 7.1	2006–2020
Companion	15.9	23.8	3.9^	2.5; 5.4	2006–2020
Regular consumption of fruits and vegetables *					
No	12.6	−20.9	3.7 ^	2.5; 5.0	2006–2020
Yes	11.8	23.4	5.2 ^	3.1; 7.4	2006–2020
Excessive consumption of sugary drinks **					
No	12.1	20.2	3.8 ^	2.4; 5.2	2006–2020
Yes	13.4	28.0	4.7 ^	1.6; 7.9	2006–2020
**TV time per day on 5 days a week**					
Does not watch or watches up to 3 h	13.3	19.3	3.5^	2.3; 4.6	2006–2020
Watches 3 or more hours	10.2	28.9	6.2^	4.0; 8.4	2006–2020

* Regular consumption of fruits, vegetables and legumes on five or more days a week. ** Consumption of soft drinks and artificial juices with sugar on five or more days a week. ^ APC is significantly different from zero at the alpha = 0.05 level.

## Data Availability

Data presented in this study are publicly and unrestrictedly available by the Brazilian Ministry of Health on the website of the IT Department of Unified Health System. https://datasus.saude.gov.br/ (accessed on 10 October 2021).
